# Diabetes-associated Genetic Variation in *MTNR1B* and Its Effect on Islet Function

**DOI:** 10.1210/jendso/bvae130

**Published:** 2024-07-09

**Authors:** Max Vella, Sneha Mohan, Hannah Christie, Kent R Bailey, Claudio Cobelli, Chiara Dalla Man, Aleksey Matveyenko, Aoife M Egan, Adrian Vella

**Affiliations:** Division of Endocrinology, Diabetes & Metabolism, Mayo Clinic College of Medicine, Rochester, MN 55905, USA; Division of Endocrinology, Diabetes & Metabolism, Mayo Clinic College of Medicine, Rochester, MN 55905, USA; Division of Endocrinology, Diabetes & Metabolism, Mayo Clinic College of Medicine, Rochester, MN 55905, USA; Division of Biomedical Statistics and Informatics, Mayo Clinic, Rochester, MN 55905, USA; Department of Women and Children's Health, University of Padova, 35128 Padova, Italy; Department of Information Engineering, University of Padova, 35128 Padova, Italy; Department of Physiology and Biomedical Engineering, Mayo Clinic, Rochester, MN 55905, USA; Division of Endocrinology, Diabetes & Metabolism, Mayo Clinic College of Medicine, Rochester, MN 55905, USA; Division of Endocrinology, Diabetes & Metabolism, Mayo Clinic College of Medicine, Rochester, MN 55905, USA

**Keywords:** *MTNR1B*, *TCF7L2*, beta-cell function, alpha-cell function, glucagon suppression, insulin secretion

## Abstract

**Context:**

Multiple common genetic variants have been associated with type 2 diabetes, but the mechanism by which they predispose to diabetes is incompletely understood. One such example is variation in *MTNR1B,* which implicates melatonin and its receptor in the pathogenesis of type 2 diabetes.

**Objective:**

To characterize the effect of diabetes-associated genetic variation at rs10830963 in the *MTNR1B* locus on islet function in people without type 2 diabetes.

**Design:**

The association of genetic variation at rs10830963 with glucose, insulin, C-peptide, glucagon, and indices of insulin secretion and action were tested in a cohort of 294 individuals who had previously undergone an oral glucose tolerance test (OGTT). Insulin sensitivity, β-cell responsivity to glucose, and Disposition Indices were measured using the oral minimal model.

**Setting:**

The Clinical Research and Translation Unit at Mayo Clinic, Rochester, MN.

**Participants:**

Two cohorts were utilized for this analysis: 1 cohort was recruited on the basis of prior participation in a population-based study in Olmsted County. The other cohort was recruited on the basis of *TCF7L2* genotype at rs7903146 from the Mayo Biobank.

**Intervention:**

Two-hour, 7-sample OGTT.

**Main Outcome Measures:**

Fasting, nadir, and integrated glucagon concentrations.

**Results:**

One or 2 copies of the G-allele at rs10830963 were associated with increased postchallenge glucose and glucagon concentrations compared to subjects with the CC genotype.

**Conclusion:**

The effects of rs10830963 on glucose homeostasis and predisposition to type 2 diabetes are likely to be partially mediated through changes in α-cell function.

The application of genome-wide association scans to the study of type 2 diabetes (T2DM) has identified multiple common genetic variants regulating pathways not previously associated with the pathogenesis of diabetes [1]. In certain circumstances, these variants can be used as probes to understand their contribution to glucose homeostasis prior to the development of diabetes. For example, the single nucleotide polymorphism (SNP) at rs7903146 in the *TCF7L2* locus is associated with impaired α-cell function [2, 3], implicating glucagon abnormalities in the early pathogenesis of prediabetes. Another insight provided by genome-wide association scans is the observation that some variants are associated with increased fasting glucose but not with T2DM risk. Other variants are associated with both increases in fasting glucose and T2DM risk while others have no association with fasting glucose but increase T2DM risk [4]. This implies differential regulation of fasting and postprandial glucose.

Genetic variation in *MTNR1B,* which encodes the melatonin receptor 2, is associated with T2DM. rs1387153, a SNP approximately 28.3 kb upstream of *MTNR1B*, is associated with increased fasting glucose and T2DM risk [5]. This SNP is in linkage disequilibrium (r^2^ = 0.7) with an intronic SNP (rs10830963) shown in other independent cohorts to be associated with fasting glucose, T2DM, and reduced β-cell function [6-8]. This SNP has also been associated with gestational diabetes [9]. Melatonin is an important component of circadian regulation, and its concentrations are highest when insulin secretion is lowest [10]. Circadian disruption and shift work have been associated with increased T2DM risk and impaired insulin secretion [11, 12]. Melatonin prevents these deleterious effects and promotes β-cell survival and function in isolated human islets and rodent models of circadian disruption [13, 14]. Moreover, low nocturnal melatonin levels are associated with increased risk of T2DM in humans [15]. However, it is also important to note that some studies demonstrated that acute administration of melatonin in humans worsens glucose tolerance, an effect exacerbated in people with 1 or more of the diabetes-associated allele at rs10830963. These somewhat conflicting observations have been described as the “melatonin paradox” emphasizing the importance of additional investigations into melatonin's effects on human islet function [16, 17].

The effect of rs10830963 on fasting glucose seems to be greater than its effects on T2DM predisposition [7]. It does not seem to be associated with glucose intolerance and has effects on insulin secretion that are apparent with both oral [6, 8] and intravenous challenges [8]. However, the methodologies used to measure β-cell function in the large cohorts described have been somewhat qualitative and subject to some limitations [18].

Recently, Heianza et al reported an analysis of this SNP in the OmniCarb trial where overweight, middle-aged adults without diabetes were randomized to a sequence of 4 complete study diets that differed in composition by carbohydrate amount and glycemic index. Participants consumed each diet for 5 weeks, with a 2-week washout between each dietary period. They report that the diabetes-associated allele increased fasting glucose and early glucose response to an oral glucose tolerance test (OGTT). However, there were no observable effects on insulin secretion as measured by the insulinogenic index. In addition, overall β-cell function quantified by a disposition index (DI) (insulinogenic index * Matsuda index) was not associated with the SNP [19].

These results are intriguing for several reasons. We have recently shown that impaired fasting glucose is caused in part by impaired suppression of glucagon secretion by glucose. Second, the melatonin receptor 2 receptor is expressed in the α-cell and melatonin augments glucagon secretion in isolated human islets [20], suggesting that genetic variation in *MTNR1B* could alter glucagon secretion [21]. Finally, the lack of any meaningful change in insulin secretion to explain the observed postchallenge hyperglycemia might also be attributable to α-cell dysfunction.

We therefore used our database [2, 22] to compare subjects homozygous for the diabetes-protective allele (CC at rs10830963) with those having 1 or 2 copies of the diabetes-associated allele (CG/GG at rs10830963) in *MTNR1B*. We report that there was a small but significant decrease in the dynamic component of β-cell responsivity accompanied by increased fasting and postchallenge glucagon concentrations in people with the CG or GG genotype. Since genotype at rs7903146 was known in these cohorts, we also show that diabetes-associated genetic variation in *TCF7L2* seems to have a greater effect on raising peak glucose concentrations as well as impairing the suppression of postchallenge glucagon concentrations.

## Methods

### Screening

As part of 2 prior published studies [2, 22] approved by the Mayo Clinic Institutional Review Board, potentially eligible subjects who expressed interest in participating were invited to the Clinical Research and Trials Unit for a screening visit. One cohort was identified after participation in a prior population-based cohort study, while the other group was recruited on the basis of *TCF7L2* genotype (either CC or TT at rs7903146 but the groups were matched for age, weight, sex, and fasting glucose). After written, informed consent was obtained, subjects underwent a history and physical examination with relevant laboratory testing. This ensured that subjects fulfilled the inclusion criteria for the studies. Body composition was measured at the time of screening using dual-energy X-ray absorptiometry (Lunar, Madison, WI). A 7-sample OGTT was completed on the day of screening or on a separate study day. Subject characteristics are reported in Table 1.

**Table 1.** Subject characteristics by genotype at rs10830963

Genotype at rs10830963	CC	CG/GG	*P*-value
n	148	146	
Age (years)	57 ± 1	56 ± 1	0.58
M/F	67/81	60/86	
Weight (kg)	80 ± 1	79 ± 1	0.61
BMI (kg/M^2^)	27.5 ± 0.4	27.4 ± 0.4	0.86
LBM (kg)	46 ± 1	45 ± 1	0.31
Fasting glucose (mmol/L)	5.52 ± 0.05	5.57 ± 0.04	0.46
Peak glucose (mmol/L)	10.5 ± 0.1	11.0 ± 0.1	0.02
120-minute glucose (mmol/L)	8.3 ± 0.2	8.5 ± 0.2	0.51
Fasting insulin (pmol/L)	37 ± 2	35 ± 2	0.46
Peak insulin (pmol/L)	455 ± 28	437 ± 23	0.63
Fasting glucagon (ng/L)	76 ± 2	82 ± 2	0.02
Nadir glucagon (ng/L)	54 ± 1	58 ± 1	0.04

Abbreviations: BMI, body mass index; LBM, lean body mass.

### Experimental Design—OGTT

The OGTT was performed after an overnight fast. A dorsal hand vein was cannulated at 0900 and placed in a heated Plexiglas box maintained at 55 °C to allow sampling of arterialized venous blood. At 0900 (0 minutes) subjects ingested 75 g of glucose. Blood was collected at 0, 10, 20, 30, 60, 90, and 120 minutes to enable measurement of glucose and hormone concentrations. At the end of the study (1100, 120 minutes), the cannula was removed; participants consumed lunch and left the Clinical Research and Trials Unit.

### Analytic Techniques

All blood was immediately placed on ice after collection, centrifuged at 4 °C, separated, and stored at −80 °C until assay. Plasma glucose concentrations were measured using a Yellow Springs glucose analyzer (Yellow Springs, OH). Plasma insulin concentrations were measured using a chemiluminescence assay (Access Assay, Beckman, Chaska, MN). Plasma C-peptide was measured using a 2-site immunenzymatic sandwich assay (Roche Diagnostics, Indianapolis, IN). Plasma glucagon and C-peptide were measured by Radioimmunoassay (Linco Research, St. Louis, MO). Genotyping of the rs10830963 and rs7903146 SNPs were performed using a Taqman™ kit (Applied Biosystems Inc., Foster City, CA).

## Calculations and Statistical Analysis

### Calculations

Net postprandial insulin action [insulin sensitivity (*S*_i_)] and β-Cell responsivity (Ф) were estimated using the oral minimal model and the oral C-peptide minimal model, respectively [23], incorporating age-associated changes in C-peptide kinetics [24]. DI for each subject was subsequently calculated by multiplying Ф by *S*_i_.

### Statistical Analysis

All continuous data are summarized as means ± SEM. Area under the curve (AUC) and area above basal (AAB) were calculated using the trapezoidal rule. To assess between-group differences, we used a 2-tailed Student's unpaired *t*-test (parametric) or a Wilcoxon test (nonparametric). To examine the interaction of rs7903146 genotype with rs10830963 genotype, we used 1-way ANOVA and a Tukey's post hoc test to determine between-group differences (parametric data). For nonparametric data, a Kruskal–Wallis test followed by a Dunn's post hoc test was used. Prism 8 (GraphPad Software, San Diego, CA) was utilized for the statistical analysis. A *P*-value <.05 was considered statistically significant.

## Results

### Subject Characteristics by Genotype

Genotyping all the study participants demonstrated that 148 subjects had the CC genotype at rs10830963 (Table 1). Twenty subjects had the GG genotype, and the remaining 126 had the CG genotype. The latter 2 genotypes were combined into 1 group as has been done before [19]. Age, weight, body composition, and fasting glucose did not differ between genotype groups.

### Glucose, Insulin, C-peptide, and Glucagon Concentrations by Genotype

**Figure 1.**
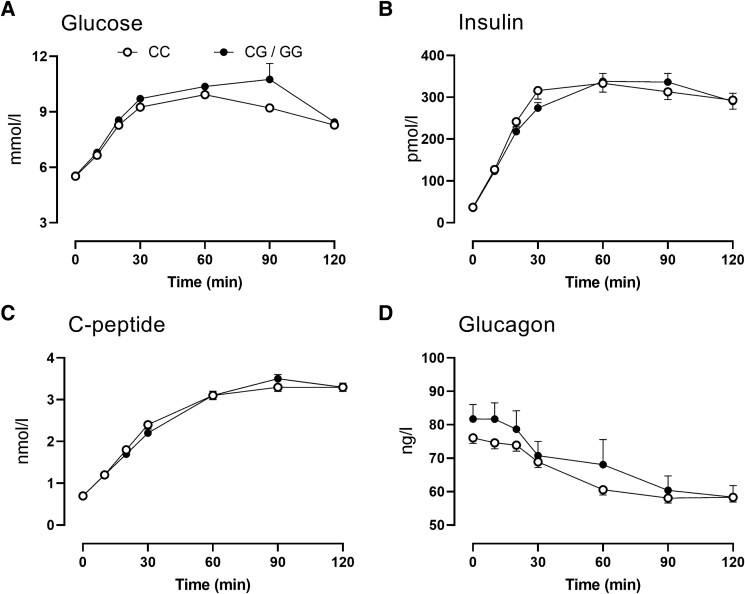
Glucose (A), insulin (B), C-peptide (C), and glucagon (D) concentrations during fasting and in response to an oral glucose tolerance test in 294 subjects with the CC (○) or the CG/GG (•) genotype at rs10830963. Values plotted are means ± SEMs.

**Figure 2.**
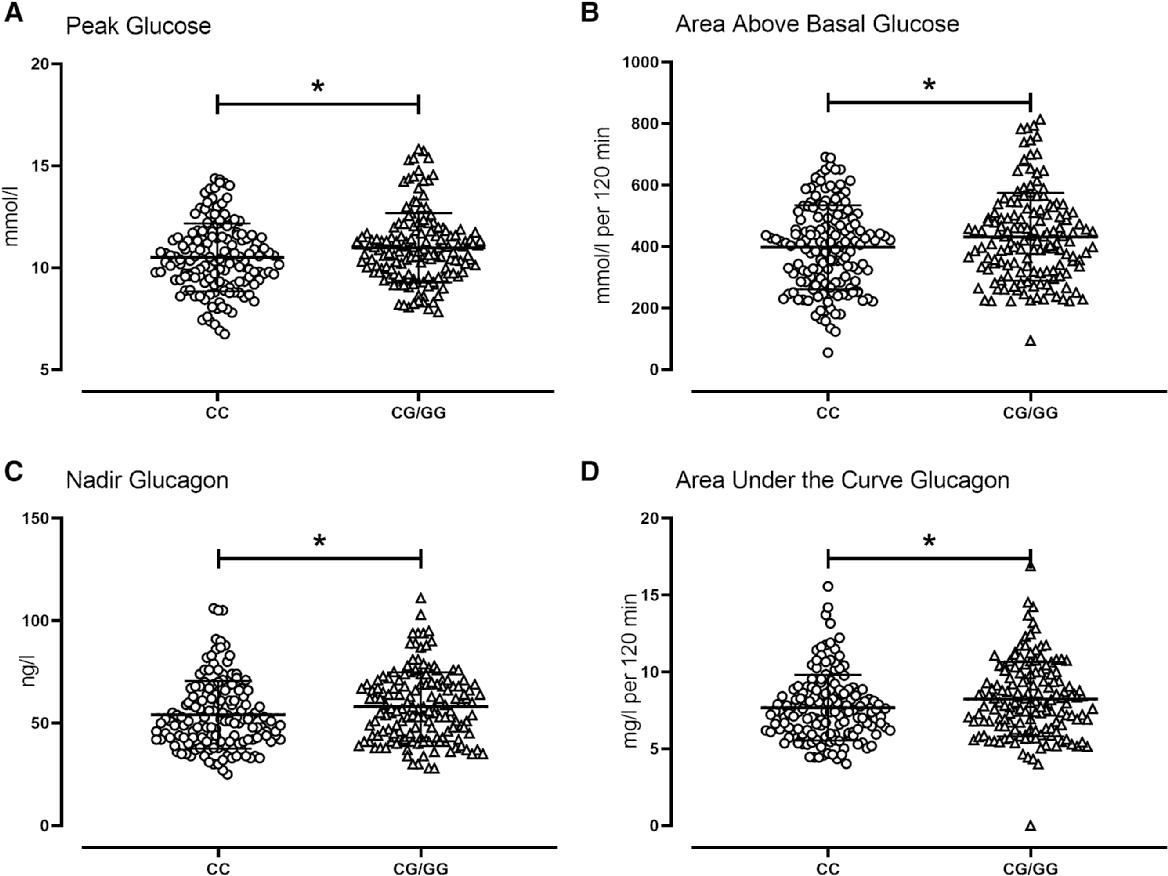
Individual values for peak glucose (A), area above basal glucose (B), nadir glucagon (C), and area under the curve glucagon (D) after oral glucose tolerance test in 294 subjects with the CC (○) or the CG/GG (Δ) genotype at rs10830963. The bars and error bars represent means ± SDs. *P < .05 for a Kruskal–Wallis test.

In people with a G-allele, peak and integrated (395 ± 11 vs 468 ± 29 mmol/L per 2 hour, *P* = .02) glucose concentrations (AAB) in response to the glucose challenge were higher than those with the CC genotype (Fig. 1A). However, at 120 minutes after the glucose challenge there were no between-group differences in glucose concentrations (please also refer to Fig. 2A and 2B).

Fasting, peak, and integrated insulin concentrations (AAB) did not differ between genotype groups (Fig. 1B). C-peptide concentrations followed the same pattern and also did not differ between groups (Fig. 1C).

In people with a G-allele, fasting glucagon concentrations were higher compared to people with the CC genotype. Nadir glucagon concentrations were also slightly, but significantly, higher (Fig. 2C). AUC glucagon concentrations over the 120 minutes postglucose challenge were also increased (Fig. 2D) in people with a G-allele (7.7 ± 0.2 vs 8.3 ± 0.2 mg/L per 2 hour, *P* = .02; Fig. 2D).

### Indices of Insulin Secretion and Action by Genotype

**Figure 3.**
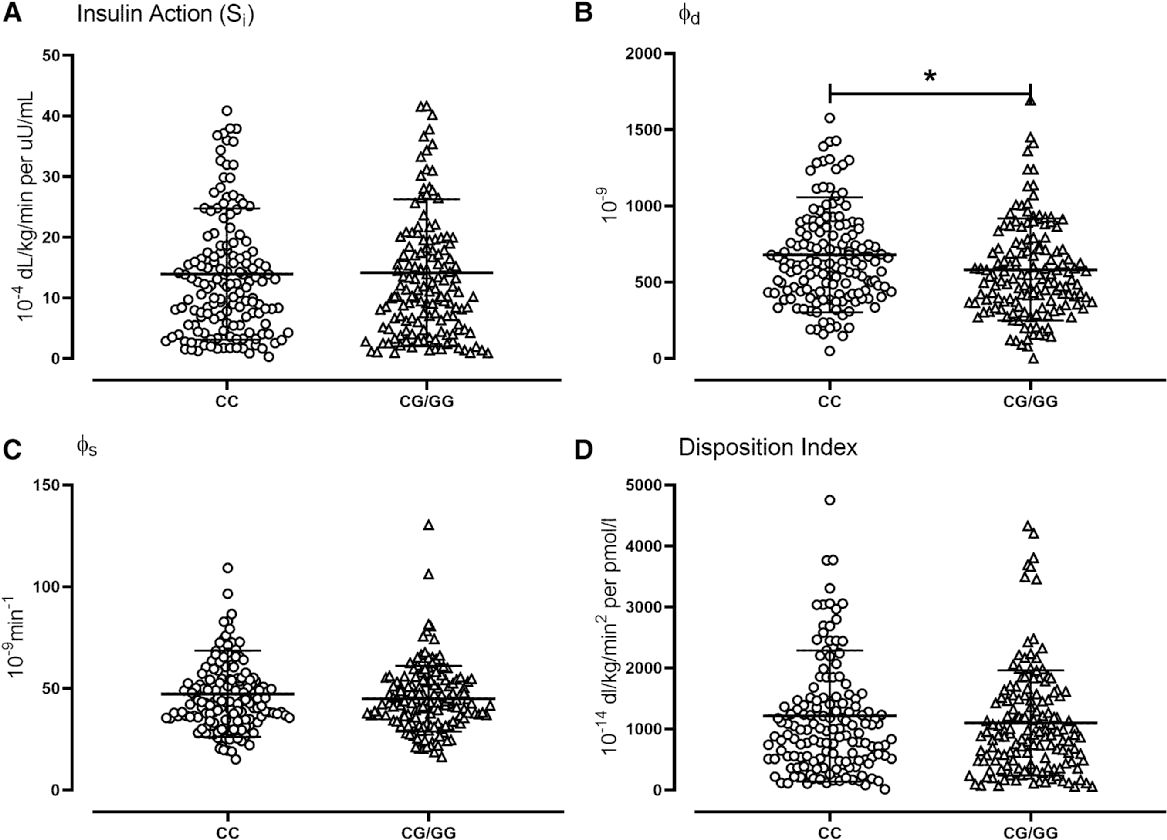
Individual values for insulin action (*S_i_*, A), the dynamic component of β-cell responsivity (ϕ_d_, B), the static component of β-cell responsivity (ϕ_s_, C), and DI (D) after oral glucose tolerance test in 294 subjects with the CC (○) or the CG/GG (Δ) genotype at rs10830963. The bars and error bars represent means ± SDs. *P < .05 for a Kruskal–Wallis test. Abbreviations: DI, disposition index.

Insulin action (*S_i_*) in response to the oral challenge did not differ (14 ± 1 vs 14 ± 1 10^−4^ dL/kg/min per μU/mL, *P* = .86) between groups (Fig. 3A). Fasting β-cell responsivity to glucose (ϕ_b_) also did not differ (7.4 ± 0.3 vs 7.3 ± 0.3 10^−9^ minutes^−1^, *P* = .82) between groups (not shown). In contrast, the dynamic component of β-cell responsivity to glucose (ϕ_d_) was decreased in people with a G-allele compared to people with the CC genotype (681 ± 31 vs 583 ± 28 10^−9^, *P* = .02; Fig. 3B).

The static component of β-cell responsivity to glucose (ϕ_s_) did not differ between genotype groups (47 ± 2 vs 45 ± 1 10^−9^ minutes^−1^, *P* = .27; Fig. 3C). Total β-cell responsivity to glucose (Φ—55 ± 2 vs 51 ± 1 10^−9^ minutes^−1^, *P* = .15) also did not differ between genotype groups (not shown). The DI also did not differ significantly (1215 ± 89 vs 1099 ± 71 10^−14^ dL/kg/min^2^ per pmol/L, *P* = .31) between genotype groups (Fig. 3D).

### Interaction of Hormone and Substrate Concentrations by MTNR1b (rs10830963) and TCF7L2 (rs7903146) Genotype

We examined the effect of the G-allele at rs10830963 on hormone and substrate concentrations in the people with 2 copies of the diabetes-protective allele (CC) and those with 2 copies of the diabetes-associated allele (TT) in the *TCF7L2* locus.

**Figure 4.**
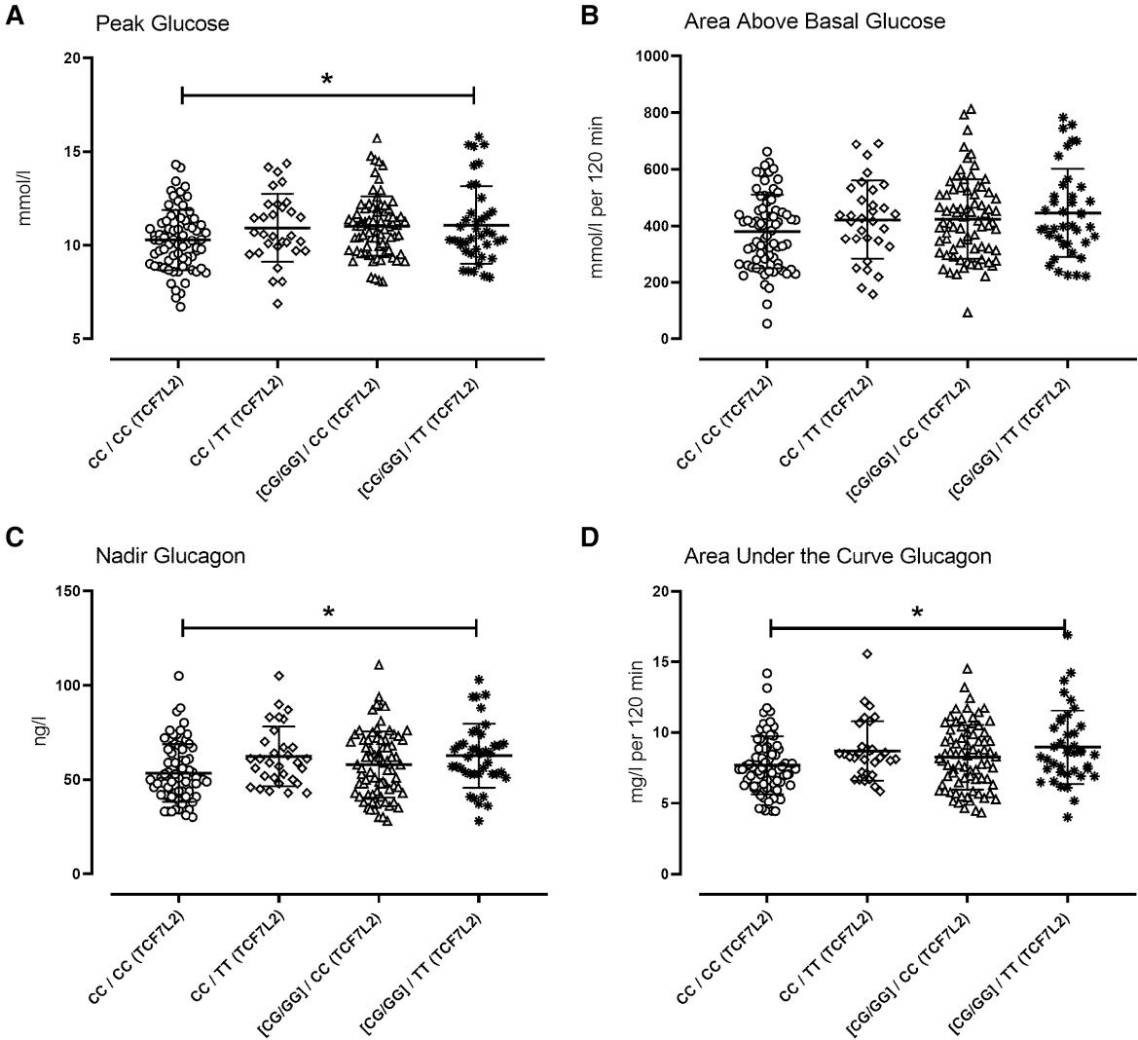
Individual values for peak glucose (A), area above basal glucose (B), nadir glucagon (C), and area under the curve glucagon (D), respectively, in people with the CC genotype at rs10830963 and rs7903146 (○, n = 73); the CC genotype at rs10830963 and the TT genotype at rs7903146 (◊, n = 31); the CG/GG genotype at rs10830963 and the CC genotype at rs7903146 (Δ, n = 76); the CG/GG genotype at rs10830963 and the TT genotype at rs7903146 (Δ, n = 40). The bars and error bars represent means ± SDs. *P < .05 for 1-way ANOVA.

Fasting glucose did not differ between genotype groups. Peak postchallenge glucose concentrations differed (*P* = .03) among the 4 groups with and without diabetes-associated variation at either SNP (Fig. 4A—10.3 ± 1.6 vs 10.9 ± 1.7 vs 11.1 ± 1.6 vs 11.0 ± 1.9 mmol/L; CC/CC vs CC/TT vs GX/CC vs GX/TT respectively—rs10830963/rs7903146). Integrated (AAB) glucose concentrations did not differ between groups (Fig. 4B).

Indices of insulin secretion and action did not exhibit any interaction (data not shown). Fasting glucagon also did not differ between genotype groups (data not shown). Nadir glucagon differed significantly between groups (Fig. 4C—54 ± 2 vs 62 ± 3 vs 58 ± 2 vs 60 ± 2 ng/L; *P* = .03). Integrated glucagon concentrations exhibited a similar relationship (Fig. 4D—7.7 ± 0.2 vs 8.7 ± 0.4 vs 8.2 ± 0.3 vs 8.7 ± 0.3 mg/L per 120 minutes; *P* = .04).

### Glucagon Concentrations in People Without and With Diabetes-associated Variation at Both MTNR1b (rs10830963) and TCF7L2 (rs7903146)

**Figure 5.**
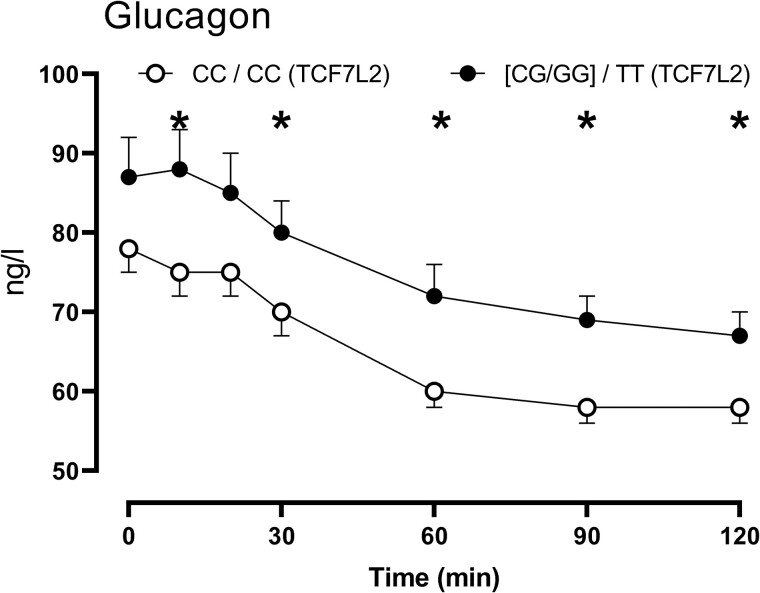
Glucagon concentrations over time in people with the CC genotype at rs10830963 and rs7903146 (○, n = 73) compared to those with the CG/GG genotype at rs10830963 and the TT genotype at rs7903146 (•, n = 40). Values plotted are means ± SEMs. *P < .05 for an unpaired, 2-tailed *t*-test.

A comparison of glucagon concentrations in people with the CC genotype at both rs10830963 and rs790146 with those who had one or two G alleles at rs1083096 and the TT genotype at rs7903146 did not demonstrate changes in fasting glucagon (78 ± 3 vs 86 ± 4 ng/L, *P* = .10). In keeping with the differences in AUC glucagon (7.7 ± 0.2 vs 8.7 ± 0.3 mg/L per 120 minutes; *P* = .03) significant differences in glucagon concentrations were observed at various timepoints (Fig. 5). This was of greater magnitude than that attributable to variation at rs10830963 (Fig. 1D) alone.

## Discussion

Postprandial glucose concentrations are dependent on the ability of the endocrine pancreas to secrete insulin in addition to the ability of insulin to suppress endogenous glucose production and to stimulate glucose uptake [23]. The ability of the α-cell to respond to hyperglycemia by suppressing glucagon secretion is also a key factor in the regulation of postprandial glucose [25]. This becomes more important as β-cell function and insulin action decrease [26]. The recent report by Heianza et al examining the effect of diabetes-associated genetic variation on glucose tolerance in the *MTNR1B* locus did not show an effect on β-cell function. This raised the possibility that effects of this variant on fasting and postprandial glucose concentrations are mediated through effects on α-cell function [19].

Previously, we showed that diabetes-associated variation in *TCF7L2* is associated with impaired α-cell function [2]. This illustrated that α-cell dysfunction arises early in the pathogenesis of T2DM. In keeping with this finding, abnormalities in glucagon secretion could predict a decline in glucose tolerance [27]. More recently, impaired fasting glucose has been shown to occur in people with intact β-cell function, and this abnormality can be explained by decreased responsiveness of the α-cell to glucagon [28].

Therefore, we examined the association of variation in rs10830963 with islet function in 2 cohorts studied using a 7-sample OGTT [2, 22]. People with 1 or 2 copies of the diabetes-associated allele at rs10830963 had higher peak and integrated postchallenge glucose concentrations. Fasting and 120-minute glucose concentrations did not differ from those observed in people without diabetes-associated variation in rs10830963. A lack of effect on 120-minute glucose is congruent with reports from several other similarly powered cohorts [8, 19].

On the other hand, we failed to observe an effect on fasting glucose. This might be explained by the fact that a subgroup of our participants was recruited on the basis of rs7903146 genotype—another SNP associated with fasting hyperglycemia. Because of the nature of that experiment, we matched fasting glucose in the cohorts with the CC and TT genotype at rs7903146. As we acknowledged at the time, this may have introduced a conservative error in our estimation of the diabetogenic effects of the T-allele [2]. Nevertheless, we did observe higher fasting glucagon in subjects with diabetes-associated variation at rs10830963.

In addition to higher fasting glucagon, the G-allele at rs10830963 was associated with impaired glucagon suppression in response to glucagon ingestion as implied by the higher nadir and integrated glucagon concentrations observed in this group. The only other accompanying defect in β-cell function that we observed was in the dynamic component of β-cell responsivity to glucose. This is thought to represent the pool of insulin granules that are primed for release in response to rising glucose concentrations [29]. Other investigators have previously suggested an effect of MTNR1B on first-phase insulin secretion in response to an intravenous glucose tolerance test, which would be in keeping with our observations [6, 8, 30].

The regulation of postprandial glucose concentrations is well characterized and depends on insulin secretion and action [23], glucose effectiveness, and glucagon suppression [25]. The rate of gastric emptying also plays a role, although this is less important in the response to an oral liquid glucose challenge [31]. In this analysis, similar time to peak glucose suggests that gastric emptying was not a factor in explaining the differences between genotype groups. Insulin secretion and action also did not differ, strongly suggesting that the differences in glucagon concentrations observed could explain postchallenge glucose. As in other studies, differences in α-cell function can occur independently of β-cell function [2, 32, 33].

Melatonin's action is mediated through high-affinity melatonin receptors [34] expressed in human islets. Persistent (6-12 hours) activation of the melatonin receptor mimicking timing of nightly exposure leads to sensitization and enhancement of adenylate cyclase activity resulting in increased activation of the cAMP, protein kinase A, and cAMP-responsive element binding protein [35]. This phenomenon also occurs in β-cells [13, 36, 37] The ability of melatonin to potentiate the cAMP-dependent signal transduction pathway is important for its actions to regulate circadian clock gene expression [36]. However, this pathway also regulates β-cell function and survival in human islets. Indeed, activation of protein kinase A is essential for glucose-stimulated insulin secretion [38]. Moreover, cAMP-responsive element binding protein expression is essential for maintaining proper β-cell mass and function and preventing β-cell apoptosis [39]. Activation of melatonin signaling in β-cells (with a duration designed to mimic typical nightly exposure) significantly enhances the cAMP-dependent signal transduction pathway and attenuates β-cell oxidative stress and apoptosis [13] restoring glucose-stimulated and incretin-stimulated insulin secretion in islets isolated from subjects with T2DM [13]. In contrast, others noted that genetic overexpression of the melatonin receptor in β-cells attenuates insulin secretion [40]. Unfortunately, much less is known about the effect of melatonin and its receptors on α-cell function.

Since *TCF7L2* genotype in both cohorts was known, we examined the effect of diabetes-associated alleles at both loci on glucose and islet function. Unsurprisingly, diabetes-associated variation at rs7903146 was associated with impaired α-cell function. The G-allele at rs10830963 did not interfere with these effects and perhaps may have a small additive effect on postchallenge glucagon concentrations when compared to the cohort. Whether this proves to be the case in larger, independent cohorts remains to be ascertained. If so, it would imply independent mechanisms of the action of *TCF7L2* and *MTNRB1* on α-cell function.

This study has several limitations, in part arising from its retrospective nature. The potential effect of how 1 cohort was recruited [2] has already been discussed. In addition, the relatively small sample size precludes a better analysis of the interaction, if any, between diabetes-associated variation at rs7903146 and rs10830963. It likely explains why we did not see an effect on β-cell function previously observed in larger studies [6, 8], although it implies that effects on α-cell function are easier to detect than effects on β-cell function. Finally, the glucagon assay used in these studies cross-reacts with other proglucagon-derived peptide fragments [41]. This raises the possibility that genetic variation in *MTNR1B* alters concentrations of proglucagon peptides other than glucagon. However, this would not explain the hyperglycemia we and others observed in the absence of significant differences in β-cell function. Despite differences in absolute concentrations of fasting and nadir glucagon measured using newer assays, there is good correlation between these assays and the assay used in this experiment [41-44]. Moreover, the association of the T-allele at rs7903146 with higher glucagon concentrations has been observed using other immunoassays in addition to this one [3].

In conclusion, we demonstrate that diabetes-associated genetic variation in *MTNR1B* can raise glucose concentrations through α-cell dysfunction in addition to having effects on insulin secretion. Future studies in larger cohorts should be better able to characterize an interaction, if any, with diabetes-associated variation at the *TCF7L2* locus.

## Data Availability

The datasets generated during and/or analyzed during the current study are available from the corresponding author upon reasonable request. No applicable resources were generated or analyzed during the current study.
